# Applications of arterial stiffness markers in peripheral arterial disease

**DOI:** 10.1590/1677-5449.009318

**Published:** 2019-03-06

**Authors:** Daniel Mendes-Pinto, Maria da Glória Rodrigues-Machado

**Affiliations:** 1 Departamento de Cirurgia Vascular, Hospital Felício Rocho, Belo Horizonte, MG, Brasil.; 2 Faculdade Ciências Médicas de Minas Gerais – FCMMG, Belo Horizonte, MG, Brasil.

**Keywords:** arterial stiffness, peripheral arterial disease, pulse wave velocity

## Abstract

Arterial stiffness has been analyzed in many different population groups with the objective of identifying cardiovascular risk early and performing specific therapeutic interventions. Increased arterial stiffness affects the capacity of the aorta and elastic arteries to adapt to pressure variations during the cardiac cycle. The main markers of arterial stiffness are pulse wave velocity (PWV), augmentation index (AIx) and central aortic pressure. They can be measured noninvasively. Patients with coronary disease or on hemodialysis who have elevated PWV or AIx have increased mortality. The association with peripheral arterial disease has been studied little. The objective of this review is to demonstrate the applicability and utility of assessing measures of arterial stiffness in patients with peripheral arterial disease.

## INTRODUCTION

 Arterial stiffness is the term employed to describe changes to physical properties of the artery wall such as distensibility, complacency, and elasticity. The physical characteristics of the artery wall have functional implications because they affect the manner in which the arteries adapt to pressure and blood flow with each heartbeat. 1

 Increased arterial stiffness is primarily the result of arteriosclerosis, a disease of the tunica media associated with aging. 2 It is a process that is accelerated by traditional cardiovascular risk factors such as hypertension, diabetes, obesity, and dyslipidemia. 3 Arterial stiffness can increase the risk of development of arterial obstructions as a result of the hemodynamic stress associated with changes to the pattern of flow through the elastic arteries, in particular increased peak systolic pressure and reduced diastolic flow. 1

 Peripheral arterial disease (PAD) of the lower limbs is primarily caused by progression of atherosclerosis in the arteries of the limbs. The increased longevity of populations has caused the estimated number of people with PAD worldwide to increase by 23.5% over 12 years, from 164 million people in 2000 to 202 million in 2012. 4 The prevalence among men aged 60 to 64 years varies from 5.5% in low to medium income countries to 8.8% in high income countries. 4 A population study conducted in Brazil with 1,159 patients with a mean age of 43.8 years found a 10.5% prevalence of PAD. 5

 The objective of this study is to review the pathophysiologic mechanisms of arterial stiffness and their relationship with PAD, thereby providing a theoretical foundation for the clinical applications of arterial stiffness indexes. 

## PATHOPHYSIOLOGY OF ARTERIAL STIFFNESS

 Arteriosclerosis is a term for disease of the walls of arteries that means, literally, hardening of the arteries. It is a degenerative process of the tunica media of elastic arteries that occurs with aging and is exacerbated by cardiovascular risk factors. 3 The mechanism underlying this process is chronic vascular inflammation caused by mitochondrial oxidative stress in smooth muscle cells. 6 Arteriosclerosis causes thickening of the artery walls, reduced complacency, and increased rigidity. Atherosclerosis is a disorder that affects the artery lumens by formation of plaques in the tunica intima that are made up of deposits of fat, inflammatory cells, fibrous connective tissue, smooth muscle cells, and calcium. Reduction of the ankle-brachial index (ABI) and formation of plaques in the carotids are both associated with atherosclerosis. 2 Atherosclerosis is the causative mechanism of the majority of ischemic diseases, such as myocardial infarction and cerebral ischemia, and is the most common pattern of arteriosclerosis. 3

 The pathophysiologic changes that lead to increased blood vessel rigidity can be separated into passive and active components. The passive component of arterial stiffness consists of mechanical changes to the vascular wall. The active component comprises changes to endothelial function and smooth muscle tonus. 

 Changes undergone by major arteries during the cardiac cycle are part of the passive component of arterial stiffness. These changes are related to distension during systole and to elastic recoil during the diastole phase. During this process, the volume of blood ejected causes distension of large caliber arteries, in which the elastic component of the walls is fundamental. The elasticity of major arteries enables the pressure wave to be dampened, preventing the entire volume ejected from being directed to the peripheral circulation. Elastic recoil of the artery wall during diastole is responsible for maintenance of diastolic flow to many different tissues. This element is extremely important for organs such as the myocardium and the brain, because they need perfusion to be maintained throughout the cardiac cycle. 7

 The process of adaptation to the volume ejected during systole and the elastic recoil produced during diastole primarily take place in elastic arteries such as the aorta and the carotids. The same pattern takes place in organs of the mesenteric, splenic, and renal circulation, but not in a continuous manner, since flow is regulated by mediators associated with blood volume and nutritional intake. In the arteries of the limbs, maintenance of diastolic flow is reduced because of the greater peripheral vascular resistance and because these are muscular arteries, whose walls have a lower elastic content. 1

 Aging causes degeneration of the elastic fibers of the tunica media of major arteries. Microscopically, there is loss of internal elastic lamina, fragmentation of elastin fibers, deposition of collagen, and thickening of the media with fibrosis and calcification. 8

 Atherosclerosis increases the process of elastic degeneration of the artery wall, since it causes interruption of the continuity of the endothelium, deposition of lipids, and formation of inflammatory mediators that exacerbate the structural derangement of the vascular wall. These changes to the wall cause it to become rigid and lead to loss of the passive control mechanism consisting of reception of the stroke volume ejected during systole and elastic recoil of the wall during diastole. 

 The main elements that actively influence arterial stiffness are the tonus of the smooth musculature of smooth muscle cells and the vasodilator action of nitric oxide produced by the endothelium. In addition to these, certain factors such as advanced age and hypertension cause remodeling of the vascular smooth muscle cells, altering their phenotypical expression from contractile to synthetic. As a result of these processes, the contractility of the vascular wall is lost because of degradation of elastin and collagen abnormalities. 7 ^,^ 9 Nitric oxide causes vasodilation and relaxation of smooth muscle cells. As its availability reduces with aging and atherosclerosis, tonus increases and artery caliber decreases. 10 In addition to these aspects, other inflammatory markers and oxidation products contribute to cellular and extracellular changes that affect arterial compliance. 

## ARTERIAL STIFFNESS INDEXES

**Figure 1:**
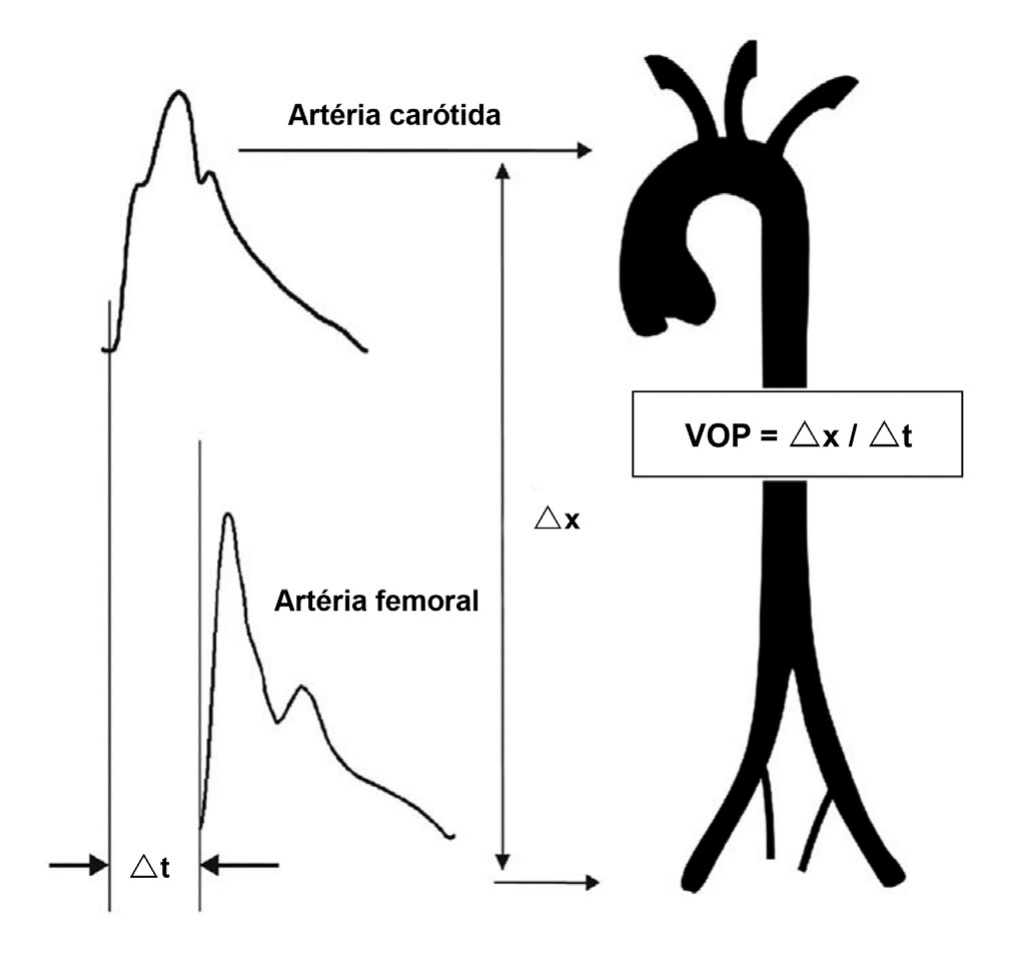
Difference between pulse waves in the carotid and common femoral arteries and calculation of the carotid-femoral pulse wave velocity. PWV: pulse wave velocity; △x: distance between two points in the arterial system; △t: time taken for the pulse wave to travel this distance. Adapted from Laurent 2

 After ventricular contraction, the pressure generated in the aorta travels like a wave. 7 Pulse wave velocity (PWV) is calculated by dividing the distance between two points in the arterial system by the time taken for the wave to travel this distance ( Figure 1 ). Pulse wave velocity measured between the carotid artery and the femoral artery is considered a predictor of cardiovascular risk and is the primary indicator of arterial stiffness. 1 In arteries with rigid walls, PWV is elevated, whereas in elastic aortas, free from atherosclerotic disease, PWV is low, in the range of 3 to 5 m/s. This is because of distension of the aorta walls. This process acts as an elastic reservoir for the stroke volume, in an effect known as *Windkessel*, which, in free translation from the German, means “air chamber”. The concept was developed by Otto Frank, a German physiologist, who also created a mathematical model for application to large vessels, which is used, with adaptations, to this day. 11 Therefore, the pressure dampening that takes place in the aorta is responsible for limiting peak systolic pressure. In turn, elastic recoil is responsible for increasing flow during diastole. 

**Figure 2:**
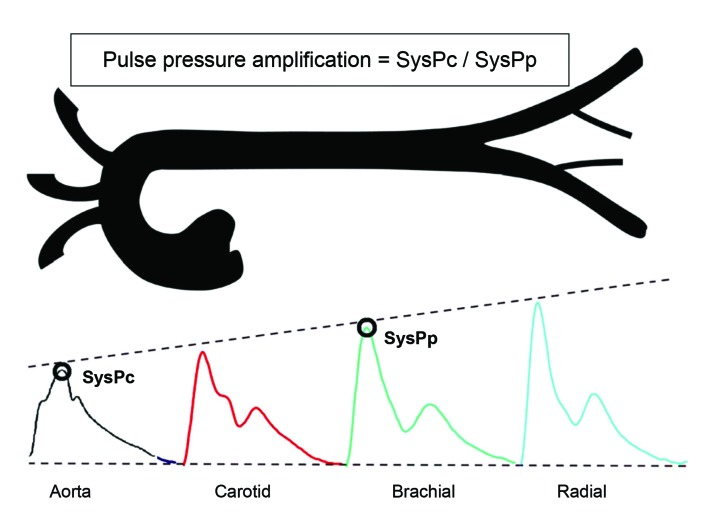
Pulse pressure amplification. Diastolic pressure and mean arterial blood pressure are relatively constant in the arterial system, but systolic pressure increases in the direction of the peripheral circulation. This phenomenon is known as pulse pressure amplification and is calculated by dividing systolic pressure central (SysPc) by peripheral systolic pressure (SysPp). Adapted from García-Espinosa et al. 14

 There are differences in blood pressure measured in the aorta and in the peripheral circulation, in, for example, the brachial artery. Systolic pressure (SysP) is lower in the central aorta, increasing as the pulse wave is transmitted to the periphery. Diastolic pressure (DiaP) increases less and may even reduce. 12 Therefore, pulse pressure (PP), which the difference between SysP and DiaP, increases in the peripheral arteries; for example, between the root of the aorta and the brachial artery, PP is amplified by around 14 mmHg. 12 ^-^ 14 This is a physiological finding known as PP amplification ( Figure 2 ). 

**Figure 3:**
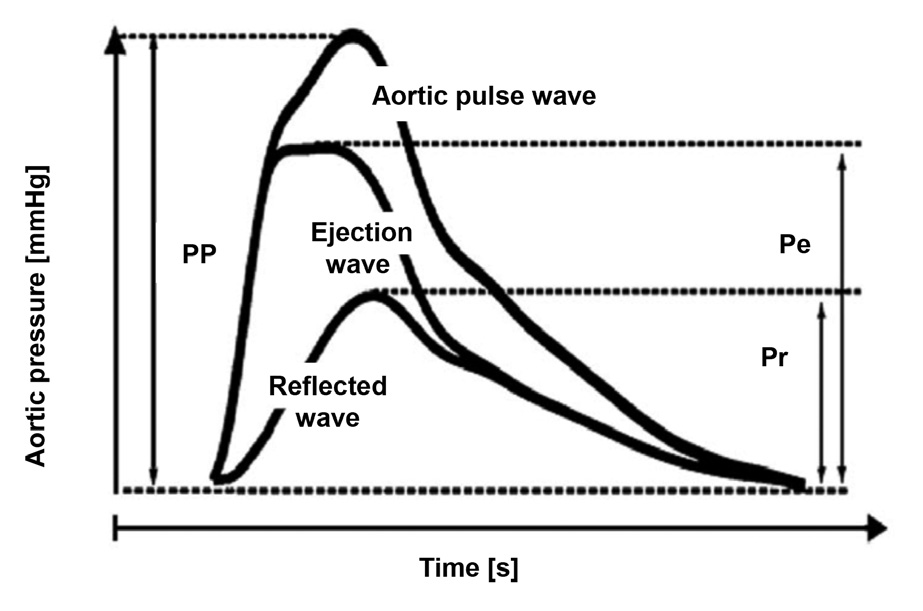
Composition of aortic pulse wave by the ejected wave and the reflected wave. The aortic pulse wave comprises the sum of the ejected wave and thee reflected wave. PP: pulse pressure. Pe: amplitude of ejected wave. Pr: amplitude of reflected wave. Adapted from García-Espinosa et al. 14

 As the pulse wave travels from the heart to the peripheral arteries, PP is amplified because of wave reflection and dampened by the viscosity of the blood. The wave of the incident pulse meets increased resistance when it reaches the peripheral muscular arteries and arterioles, causing reflection waves in the direction of the heart. These pulse reflection waves occur in any segment in which there is discontinuity of flow, such as at bifurcations and, primarily, when the incident waves reach the peripheral arteries with greater resistance and lower elasticity. Thus, to aid understanding, the waves can be designated as the incident wave (or ejection wave) and the reflected wave and the resulting pulse wave is the sum of the incident wave and the reflected wave ( Figure 3 ). 14

**Figure 4:**
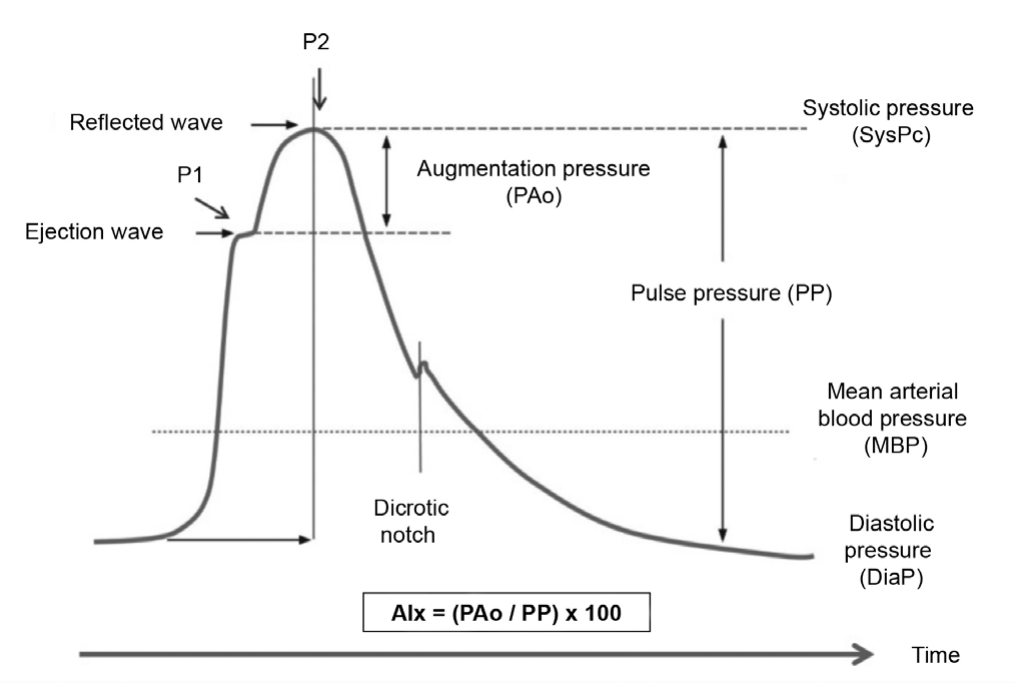
Pulse wave in a rigid proximal aorta over a cardiac cycle. The augmentation pressure (PAo) reflects the increase in pressure caused by the arrival of the reflected wave during systole. P1: systolic peak attributed to the ejected wave. P2: second systolic peak attributed to the increase in pressure caused by the reflected wave. The dicrotic notch corresponds to the final phase of ventricular ejection and is produced by closure of the aortic valve. AIx: augmentation index. Adapted from Husmann et al. 16

Figure 4 illustrates the pulse wave at the root of the aorta in a scenario of increased arterial stiffness, whether due to aging or atherosclerosis. As a result of the elevated PWV, the reflected wave arrives at the proximal aorta prematurely, at the start of systole (point P1 on the graph). This early arrival of the reflected wave causes an increase in central systolic arterial blood pressure, shown at P2 on the graph. The augmentation pressure (AuP) is a measure of the absolute increase in pressure between two systolic peaks. The augmentation index (AIx) measures the percentage of the pressure increase that is caused by the premature arrival of the reflected wave and is expressed as the ratio of PAo and PP multiplied by 100. The larger the reflected pulse waves, as in cases with elevated arteriolar tonus or arterial obstruction, the higher the AIx. Therefore, AIx is considered an indirect indicator of arterial stiffness and a predictor of cardiovascular events. 2 ^,^ 15 ^,^ 16 In a population study with 5,960 participants, each 10% increase in AIx was associated with a 1.08 relative risk of increase in the chance of major cardiovascular events, such as myocardial infarction, stroke, and even death. 17

**Figure 5:**
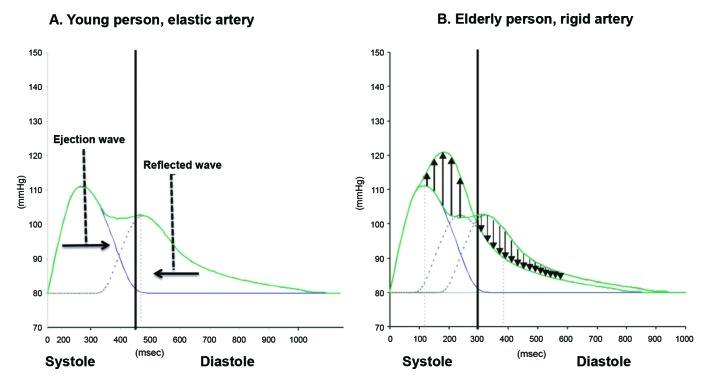
Graphs illustrating the ejected and reflected waves in the proximal aorta. **(A)** In a young person with elastic arteries, the reflected wave arrives during diastole, does not increase the systolic peak and preserves diastolic flow. **(B)** In the elderly person with a rigid aorta, the reflected wave arrives during systole, causing the systolic peak to increase and diastolic flow to reduce. Adapted from Husmann et al. 16

 Young people have high arterial elasticity. Since PWV is low in these cases, the reflected pulse wave arrives at the thoracic aorta during diastole. It therefore contributes to increase diastolic flow and maintain constant flow in the coronary and cerebral arteries. With aging, PWV increases because of the reduced elasticity of major arteries. As a result, the reflected wave arrives at the root of the aorta during systole, increasing AIx ( Figure 5 ). 

 Heart rate is an important modulator of PAo. Under low heart rate conditions, widening of the cardiac cycle and longer ejection time cause the reflected wave to arrive at the proximal aorta during systole, increasing both PAo and AIx. During tachycardia, the reflected pulse wave arrives at the root of the aorta during diastole. 16 Thus, correction of AIx for heart rate prevents this variable from having an influence on pulse wave reflection assessments. AIx@75 is AIx corrected to a heart rate of 75 beats per minute. This index is given by arterial stiffness meters and is defined as AIx@75 = Aix – 0.39 × (75 – heart rate). 18 Table 1 lists the hemodynamic parameters associated with arterial stiffness. 19

**Table 1:** Hemodynamic parameters associated with arterial stiffness (adapted from Gajdova et al.). 19

**Hemodynamic parameter**	**Calculation**	**Description**
Pulse pressure (PP, mmHg)	Systolic pressure - diastolic pressure	Difference between systolic and diastolic pressures. In peripheral arteries, PP can be up to 14 mmHg greater than central aortic pressure.
Augmentation pressure (PAo, mmHg)	P2 - P1	The difference between the second (P2) and first (P1) pressure peaks in the pulse wave. P1 corresponds to peak systolic ejection. P2 corresponds to the peak caused by arrival of the reflected pulse wave.
Augmentation index (AIx, %)	(P2 - P1)/PP x 100	AIx is derived from the difference between the second (P2) and first (P1) systolic peaks, expressed as a percentage of PP. It reflects the intensity of pulse wave reflection.
Augmentation index corrected for a heart rate of 75 bpm (AIx@75, %)	AIx@75 = AIx – 0.39 × (75 – heart rate)	AIx normalized for a heart rate of 75 bpm. An increase in heart rate reduces AIx. Bradycardia increases AIx. AIx@75 is unaltered by changes in heart rate.
Pulse wave velocity (PWV, m/s)	△x/△t	The velocity at which a pulse wave travels through the arterial system, defined as the ratio between the distance between two points in the arterial system (△x) and the time taken by the wave to cover the distance (△t). It is the most widely-used indicator of arterial stiffness because it has good reproducibility.

 In addition to heart rate, other factors modulate pulse wave reflections and central aortic pressure. Inhibition of angiotensin II, calcium channel blockers and administration of insulin reduce pulse wave reflections and central systolic pressure. 20 Peripheral resistance increased by insulin has the opposite effect, increasing reflection waves. 21

 Over recent decades, it has been shown that central pulse pressure (Cpp) is associated with increased cardiovascular risk. Both elevated Cpp and elevated peripheral pulse pressure are associated with increased risk of coronary disease in middle-aged adults and the elderly. 22 However, risk of cardiovascular events is more associated with elevated central pressures. Elevated Cpp is associated with hypertrophy of the left ventricle and carotid atherosclerosis. 15 In one study, elevated Cpp was associated with increased rates of complications and mortality in patients who underwent endovascular treatment for critical limb ischemia. 23

## REPERCUSSIONS OF INCREASED ARTERIAL STIFFNESS

 Elevated arterial stiffness is a current concept that is foregrounded in studies of cardiovascular diseases because it is an early predictor of arterial hypertension and atherosclerosis. It is a biomarker that has been associated with increased mortality and damage to target organs responsible for cardiac diseases, stroke, and renal failure. There is currently considerable research interest in investigation of arterial stiffness as a potential therapeutic target for prevention or treatment of cardiovascular diseases. 

 Many different studies have demonstrated that increased PWV is associated with increased risk of cardiovascular events such as coronary disease, stroke, and terminal kidney disease. 24 ^,^ 25

 The association between arterial stiffness and hypertension has been studied in depth. Especially in young people, identification of increased arterial stiffness is an early opportunity to modify lifestyle habits and prevent irreversible deterioration of blood vessels. 25 Analysis of the Framingham study indicated that elevated arterial stiffness, measured in terms of carotid-femoral PWV, was associated with the incidence of arterial hypertension 7 years after the initial measurement taken by researchers. 26

 Arterial stiffness increases afterload on the left ventricle, contributes to ventricular remodeling, and reduces its mechanical efficiency. 27 This increases myocardial demand and reduces diastolic perfusion. Thus, arterial stiffness is associated with diastolic ventricular dysfunction, which increases filling pressures and overload on the atria, contributing to hypertrophy, fibrosis, and atrial fibrillation. It is important to highlight that arterial stiffness is directly associated with increased risk of heart failure. 28

 The kidneys are organs that are exposed to high blood flow, because the pressures at the level of the glomerulus are similar to those in the aorta, due to the characteristics of renal microvasculature. Increased aortic rigidity exposes the renal glomerulus to excessive pressures and, over time, leads to proteinuria and impaired filtration function. 29 Several studies have demonstrated the relationship between increased PP and PWV with reduced renal function and increased mortality. 30 ^,^ 31

 In common with the kidneys, the brain is a high-flow organ and is dependent on diastolic flow. The low resistance of cerebral arteries facilitates penetration of pulse waves to the microvasculature. In this case, transmission of the pulse wave energy, which is increased in situations of elevated arterial stiffness, contributes to cerebral microinfarcts that are not recognized clinically, but which, over the long term, can lead to cognitive deficits and dementia. 32 Thus, the reduction of diastolic flow that occurs in advanced arterial stiffness contributes to brain damage. The same mechanism that increases pulsatility in cerebral arteries also links aortic rigidity to hemorrhagic strokes. 33

## METHODS FOR MEASUREMENT OF ARTERIAL STIFFNESS INDEXES

 It is possible to measure PWV using catheters in the aorta, but invasive measurement has little practical applicability. Studies employing central pressure measurements are only conducted in humans in clinical trial settings and for validation of noninvasive methods. 1

 Measurements of arterial stiffness and inference of central pressure levels are performed using mathematical models. Equipment used to determine the measurements for rigidity indexes can be classified into four categories: devices that employ tonometry to measure pulse waves, devices with cuffs that capture the pulse wave by oscillometry, ultrasound-based measurements, and measurements using magnetic resonance imaging (MRI). 

### Devices that employ tonometry to measure pulse waves

 SphygmoCor® (AtCor Medical, West Ryde, Australia) is a system that is widely used in research to measure PWV. The instrument employs a probe to measure pressure (tonometry) at two sites; generally over the cervical carotid artery and the femoral artery at the inguinal level, where the pulse is detectable. The device is synchronized using an electrocardiogram, and pulse waves are measured throughout the cardiac cycle. Distance is measured manually from the suprasternal angle to the femoral artery. 2 Other devices that have been validated for pulse wave measurement by tonometry are Complior® (ALAM Medical, Vincennes, France) and PulsePen® (DiaTecne, Milan, Italy). 1

### Devices with cuffs that capture the pulse wave by oscillometry

 The Mobil-O-Graph ® (IEM, Stolberg, Germany) estimates central aortic pressure and PWV by means of an algorithm obtained from measurements taken at the brachial artery. After measuring arterial blood pressure, the cuff is inflated to diastolic pressure for approximately 10 seconds to capture pulse waves. A mathematical model is then used to estimate a series of hemodynamic parameters, including measures of reflection waves, pressure, and AIx@75. The device has been validated for measurement of PWV by comparison with invasive and noninvasive tests. 34 ^,^ 35 It is important to mention that portable oscillometric devices offer the advantage of being less operator dependent and easier to use. Other oscillometric devices include the VP1000® (Omron Healthcare, Kyoto, Japan) and the Vasera® (Fukuda Denshi, Tokyo, Japan). 1

### Ultrasound-base measurement

 The distensibility of the arteries and the characteristics of flow curves can be easily accessed at the cervical carotid, the brachial artery, and the femoral artery, in the groin. Computer programs for measurement of the distention of vessels have been written to enable calculation of PP and estimation of central pressures (for example, ARTLAB®, Esaote, Gênova, Italy). In turn, PWV can be measured using two transducers, one over the carotid artery and the other on the abdomen, over the aorta, or over the femoral artery. In this case, the ultrasound machine must be connected to an electrocardiogram to achieve correct synchronization with the cardiac cycle and because of this the method is considered impractical and is rarely employed in clinical research. 1

### Measurement using magnetic resonance imaging

 As with ultrasound, MRI can be used to measure distensibility of the arteries as the cardiac cycle progresses, with the advantage that it can access thoracic vessels. Intravenous contrast MRI can be used to measure flow velocity and PWV. However, the costs of this method and difficulties linked to the need for trained operators limit its clinical applicability. 1

##  RELATIONSHIP BETWEEN PERIPHERAL ARTERIAL DISEASE AND ARTERIAL STIFFNESS 

 Critical ischemia, as an advanced form of PAD, can be considered a terminal stage of elevated arterial stiffness. 16 It is well known that ABI is an independent marker of mortality and morbidity, but its relationships with arterial stiffness indexes has been studied little. 36

 Elevated aortic rigidity is associated with development of atherosclerosis as a result of changes affecting flow through major arteries and shear forces during systole. 1 ^,^ 26 Aortic wall rigidity provokes a reduction in the impedance gradient (resistance to blood flow) between the complacent aorta and the peripheral circulation made up of muscular arteries. The reduced complacency of the aorta causes excessive transmission of pressure waves to the microvasculature, injuring target organs. 26 ^,^ 32

 Increased arterial stiffness is associated with shorter walking distance among patients with claudication. 37 While several publications have investigated arterial stiffness indexes in patients with claudication, 38 ^-^ 42 few have investigated patients with critical limb ischemia and the findings of those that have are inconclusive. In cases of severe aortoiliac obstruction, PWV measured at the femoral artery is reduced. 43 In contrast, the magnitude of pulse wave reflection, measured as AIx, is increased in patients with peripheral arterial disease. 44 ^,^ 45

 Arterial obstructions along the length of aortoiliac and infrainguinal segments are points of early reflection of pulse waves, while premature return to the root of the aorta during systole increases afterload on the left ventricle and reduces myocardial perfusion. 16 ^,^ 25 The relationship between myocardial supply and demand is altered unfavorably because of increased arterial stiffness and is one of the factors that increases mortality among patients with PAD. 36

 The major causes of mortality of patients with advanced PAD are ischemic heart disease, stroke, and reduced mobility because of major and minor amputations. Predictors of these outcomes have been established: advanced age, diabetes, and reduction of the ABI. 16 It is possible that indicators of arterial stiffness such as PWV and AIx could be predictors of clinical outcomes in PAD, but this relationship has not yet been studied. Knowledge of hemodynamic parameters in advanced PAD could improve understanding of the relationships between these parameters and mortality from coronary and cerebral diseases among patients with PAD. 

## CONCLUSIONS

 It is important to identify which PAD patients are at greatest risk. It is possible that elevated arterial stiffness is a factor in increased mortality among patients with claudication or critical ischemia. Along the same lines, arterial stiffness indexes may be associated with outcomes such as limb salvage or major amputations. These associations have not yet been studied. Once the relationships between the degree of ischemia in a limb and arterial stiffness indexes has been established, they could be used in longitudinal studies as prognostic factors of PAD outcomes. 

## References

[1] Townsend RR, Wilkinson IB, Schiffrin EL (2015). Recommendations for improving and standardizing vascular research on arterial stiffness: A scientific statement from the American Heart Association. Hypertension.

[2] Laurent S, Cockcroft J, Van Bortel L (2006). Expert consensus document on arterial stiffness: methodological issues and clinical applications. Eur Heart J.

[3] Fan X, Zhu M, Chi C (2017). Association of arteriosclerosis and/or atherosclerosis with hypertensive target organ damage in the community-dwelling elderly Chinese: the Northern Shanghai Study. Clin Interv Aging.

[4] Fowkes FGR, Rudan D, Rudan I (2013). Comparison of global estimates of prevalence and risk factors for peripheral artery disease in 2000 and 2010: A systematic review and analysis. Lancet.

[5] Makdisse M, Pereira AC, Brasil DP (2008). Prevalence and risk factors associated with peripheral arterial disease in the Hearts of Brazil Project. Arq Bras Cardiol.

[6] Mozos I, Malainer C, Horbańczuk J (2017). Inflammatory Markers for Arterial Stiffness in Cardiovascular Diseases. Front Immunol.

[7] Avolio A (2013). Arterial Stiffness. Pulse.

[8] Lusis AJ (2000). Atherosclerosis. Nature.

[9] Brozovich FV, Nicholson CJ, Degen CV, Gao YZ, Aggarwal M, Morgan KG (2016). Mechanisms of Vascular Smooth Muscle Contraction and the Basis for Pharmacologic Treatment of Smooth Muscle Disorders. Pharmacol Rev.

[10] Maksuti E, Westerhof N, Westerhof BE, Broome M, Stergiopulos N (2016). Contribution of the Arterial System and the Heart to Blood Pressure during Normal Aging - A Simulation Study. PLoS One.

[11] Safar ME, Levy BI, Struijker-Boudier H (2003). Current perspectives on arterial stiffness and pulse pressure in hypertension and cardiovascular diseases. Circulation.

[12] Yannoutsos A, Ahouah M, Tubiana CD (2016). Hemodynamic parameters in hypertensive diabetic patients. J Hypertens.

[13] Safar ME, Plante GE, Mimran A (2015). Arterial stiffness, pulse pressure, and the kidney. Am J Hypertens.

[14] García-Espinosa V, Curcio S, Marotta M (2016). Changes in central aortic pressure levels, wave components and determinants associated with high peripheral blood pressure states in childhood: Analysis of hypertensive phenotype. Pediatr Cardiol.

[15] Chirinos JA, Kips JG, Jacobs DR (2012). Arterial wave reflections and incident cardiovascular events and heart failure: MESA (Multiethnic Study of Atherosclerosis). J Am Coll Cardiol.

[16] Husmann M, Jacomella V, Thalhammer C, Amann-Vesti BR (2015). Markers of arterial stiffness in peripheral arterial disease. Vasa.

[17] Manisty CH, Hughes AD (2013). Meta-analysis of the comparative effects of different classes of antihypertensive agents on brachial and central systolic blood pressure, and augmentation index. Br J Clin Pharmacol.

[18] Nunan D, Wassertheurer S, Lasserson D (2012). Assessment of central haemomodynamics from a brachial cuff in a community setting. BMC Cardiovasc Disord.

[19] Gajdova J, Karasek D, Goldmannova D (2017). Pulse wave analysis and diabetes mellitus. A systematic review. Biomed Pap..

[20] Tamminen MK, Westerbacka J, Vehkavaara S, Yki-Jarvinen H (2003). Insulin therapy improves insulin actions on glucose metabolism and aortic wave reflection in type 2 diabetic patients. Eur J Clin Invest.

[21] Weber T, Wassertheurer S, Rammer M, Haiden A, Hametner B, Eber B (2012). Wave reflections, assessed with a novel method for pulse wave separation, are associated with end-organ damage and clinical outcomes. Hypertens.

[22] Zettervall SL, Buck DB, Darling JD, Lee V, Schermerhorn ML, Guzman RJ (2016). Increased preoperative pulse pressure predicts procedural complications and mortality in patients who undergo tibial interventions for critical limb ischemia. J Vasc Surg.

[23] Vlachopoulos C, Aznaouridis K, Stefanadis C (2010). Prediction of cardiovascular events and all-cause mortality with arterial stiffness: a systematic review and meta-analysis. J Am Coll Cardiol.

[24] Ben-Shlomo Y, Spears M, Boustred C (2014). Aortic pulse wave velocity improves cardiovascular event prediction: an individual participant meta-analysis of prospective observational data from 17,635 subjects. J Am Coll Cardiol.

[25] Kaess BM, Rong J, Larson MG (2012). Aortic stiffness, blood pressure progression, and incident hypertension. JAMA.

[26] Mitchell GF, Hwang S-J, Vasan RS (2010). Arterial stiffness and cardiovascular events: the Framingham Heart Study. Circulation.

[27] Mitchell GF, Tardif JC, Arnold JM (2001). Pulsatile hemodynamics in congestive heart failure. Hypertens.

[28] Hashimoto J, Ito S (2011). Central pulse pressure and aortic stiffness determine renal hemodynamics: pathophysiological implication for microalbuminuria in hypertension. Hypertens.

[29] Williams B, Lacy PS, Thom SM (2006). Differential impact of blood pressure-lowering drugs on central aortic pressure and clinical outcomes: principal results of the Conduit Artery Function Evaluation (CAFE) study. Circulation.

[30] Baumann M, Wassertheurer S, Suttmann Y, Burkhardt K, Heemann U (2014). Aortic pulse wave velocity predicts mortality in chronic kidney disease stages 2-4. J Hypertens.

[31] Mitchell GF, van Buchem MA, Sigurdsson S (2011). Arterial stiffness, pressure and flow pulsatility and brain structure and function: the Age, Gene/Environment Susceptibility-Reykjavik study. Brain.

[32] Kinjo Y, Ishida A, Kinjo K, Ohya Y (2016). A high normal ankle-brachial index combined with a high pulse wave velocity is associated with cerebral microbleeds. J Hypertens.

[33] Vlachopoulos C, Xaplanteris P, Aboyans V (2015). The role of vascular biomarkers for primary and secondary prevention. A position paper from the European Society of Cardiology Working Group on peripheral circulation. Endorsed by the Association for Research into Arterial Structure and Physiology (ARTERY). Atherosclerosis.

[34] Weiss W, Gohlisch C, Harsch-Gladisch C, Tölle M, Zidek W, Van Der Giet M (2012). Oscillometric estimation of central blood pressure: Validation of the Mobil-O-Graph in comparison with the SphygmoCor device. Blood Press Monit.

[35] Papaioannou TG, Argyris A, Protogerou AD (2013). Non-invasive 24 hour ambulatory monitoring of aortic wave reflection and arterial stiffness by a novel oscillometric device: the first feasibility and reproducibility study. Int J Cardiol.

[36] Mosimann K, Jacomella V, Thalhammer C (2012). Severity of peripheral arterial disease is associated with aortic pressure augmentation and subendocardial viability ratio. J Clin Hypertens.

[37] Brewer LC, Chai H-S, Bailey KR, Kullo IJ (2007). Measures of arterial stiffness and wave reflection are associated with walking distance in patients with peripheral arterial disease. Atherosclerosis.

[38] Catalano M, Scandale G, Carzaniga G (2013). Increased aortic stiffness and related factors in patients with peripheral arterial disease. J Clin Hypertens.

[39] Catalano M, Scandale G, Carzaniga G (2014). Aortic augmentation index in patients with peripheral arterial disease. J Clin Hypertens.

[40] Zahner GJ, Gruendl MA, Spaulding KA (2017). Association between arterial stiffness and peripheral artery disease as measured by radial artery tonometry. J Vasc Surg.

[41] Zahner GJ, Spaulding KA, Ramirez JL (2018). Characterizing the relationship between flow-mediated vasodilation and radial artery tonometry in peripheral artery disease. J Surg Res.

[42] Jacomella V, Shenoy A, Mosimann K, Kohler MK, Amann-Vesti B, Husmann M (2013). The impact of endovascular lower-limb revascularisation on the aortic augmentation index in patients with peripheral arterial disease. Eur J Vasc Endovasc Surg.

[43] Brand M, Woodiwiss AJ, Michel F, Booysen HL, Veller MG, Norton GR (2013). A mismatch between aortic pulse pressure and pulse wave velocity predicts advanced peripheral arterial disease. Eur J Vasc Endovasc Surg.

[44] Khaleghi M, Kullo IJ (2007). Aortic augmentation index is associated with the ankle-brachial index: A community-based study. Atherosclerosis.

[45] Eldrup N, Sillesen H, Prescott E, Nordestgaard BG (2006). Ankle brachial index, C-reactive protein, and central augmentation index to identify individuals with severe atherosclerosis. Eur Heart J.

