# Supplementary material for: Applications of arterial stiffness markers in peripheral arterial disease
Source: J Vasc Bras. 2019 Mar 6;18:e20180093. doi: 10.1590/1677-5449.009318 (PMC6579524; doi:10.1590/1677-5449.009318)
Supplement: Translation

# Translation: Aplicabilidade dos marcadores de rigidez arterial na doença arterial periférica

**Authors:** Daniel Mendes-Pinto, Maria da Glória Rodrigues-Machado

**Affiliations:** 1 Departamento de Cirurgia Vascular, Hospital Felício Rocho, Belo Horizonte, MG, Brasil.; 2 Faculdade Ciências Médicas de Minas Gerais – FCMMG, Belo Horizonte, MG, Brasil.

**Keywords:** rigidez arterial, doença arterial periférica, velocidade da onda de pulso

## Abstract

A análise de rigidez arterial tem sido feita em vários grupos populacionais com o objetivo de identificar precocemente o risco cardiovascular e realizar medidas terapêuticas específicas. O aumento da rigidez arterial leva à perda de capacidade de adaptação da aorta e das artérias elásticas às variações de pressão durante o ciclo cardíaco. Os principais marcadores de rigidez arterial são a velocidade de onda de pulso (VOP), o índice de aumentação (AIx) e a pressão aórtica central. Esses índices podem ser obtidos de maneira não invasiva. Ocorre aumento da mortalidade em pacientes com doença coronariana ou em hemodiálise que apresentam aumento da VOP ou do AIx. A associação com a doença arterial periférica é pouco estudada. O objetivo desta revisão é mostrar a aplicabilidade e a utilidade de realizar medidas de rigidez arterial em pacientes com doença arterial periférica.

## INTRODUÇÃO

 Rigidez arterial é o termo empregado para caracterizar as alterações de propriedades físicas da parede arterial tais como distensibilidade, complacência e elasticidade. As características físicas da parede arterial têm implicações funcionais, pois afetam a maneira como as artérias se adaptam à pressão e ao fluxo sanguíneo em cada batimento cardíaco 1 . 

 O aumento da rigidez arterial resulta primariamente da arteriosclerose, uma doença da túnica média associada ao envelhecimento 2 . É um processo que é acelerado por fatores de risco cardiovascular tradicionais como hipertensão, diabetes, obesidade e dislipidemia 3 . A rigidez arterial pode aumentar o risco de desenvolvimento de obstruções arteriais, como resultado do estresse hemodinâmico associado às alterações do padrão de fluxo nas artérias elásticas dentre as quais se destacam o aumento do pico de pressão sistólica e a redução do fluxo diastólico 1 . 

 A doença arterial periférica (DAP) nos membros inferiores é causada principalmente pela progressão da aterosclerose nas artérias dos membros. Devido ao aumento da longevidade da população, o número estimado de pessoas com DAP no mundo aumentou 23,5% em 12 anos, passando de 164 milhões de pessoas em 2000 para 202 milhões em 2012 4 . A prevalência em homens entre 60 e 64 anos varia de 5,5% em países de baixa ou média renda a 8,8% em países de alta renda 4 . Um estudo populacional feito no Brasil com 1.159 pacientes com idade média de 43,8 anos revelou que a prevalência da DAP foi de 10,5% 5 . 

 O objetivo deste estudo é rever os mecanismos fisiopatológicos da rigidez arterial e sua relação com a DAP. Dessa forma, procuramos fornecer uma base teórica para as aplicações clínicas dos índices de rigidez arterial. 

## FISIOPATOLOGIA DA RIGIDEZ ARTERIAL

 A arteriosclerose é uma doença da parede das artérias que significa, literalmente, o endurecimento das artérias. Trata-se de um processo degenerativo da túnica média das artérias elásticas que ocorre com o envelhecimento e é potencializado por fatores de risco cardiovascular 3 . O mecanismo desse processo é a inflamação vascular crônica derivada do estresse oxidativo mitocondrial na célula muscular lisa 6 . A arteriosclerose causa espessamento da parede das artérias, redução da complacência e aumento da rigidez. Aterosclerose é uma desordem que afeta o lúmen das artérias devido à formação de placas na camada íntima constituídas por depósitos de gordura, células inflamatórias, tecido conjuntivo fibroso, células musculares lisas e cálcio. A redução do índice tornozelo/braço (ITB) e a formação de placas nas carótidas estão associadas a aterosclerose 2 . A aterosclerose é o mecanismo causador da maioria das doenças isquêmicas, como infarto do miocárdio e isquemia cerebral, e constitui o padrão mais comum da arteriosclerose 3 . 

 As alterações fisiopatológicas que levam ao aumento da rigidez dos vasos sanguíneos podem ser divididas em componentes passivo e ativo. O componente passivo da rigidez arterial consiste nas alterações mecânicas da parede vascular. O componente ativo é composto pelas alterações da função endotelial e do tônus do músculo liso. 

 As alterações que as grandes artérias sofrem durante o ciclo cardíaco consistem no componente passivo da rigidez arterial. São modificações relacionadas à distensão durante a sístole e ao recolhimento elástico na fase da diástole. Nesse processo, o volume sanguíneo ejetado causa distensão das artérias de grande calibre, nas quais o componente elástico da parede é fundamental. A elasticidade das grandes artérias possibilita que a onda de pressão seja amortecida, evitando que todo o volume ejetado seja direcionado para a circulação periférica. O recolhimento elástico da parede arterial durante a diástole é responsável pela manutenção do fluxo diastólico em vários tecidos. Esse aspecto é extremamente importante em órgãos como o miocárdio e o cérebro, pois eles necessitam de manutenção da perfusão durante todo o ciclo cardíaco 7 . 

 O processo de adaptação ao volume ejetado durante a sístole e o retorno elástico produzido na fase da diástole ocorrem principalmente em artérias elásticas como a aorta e as carótidas. Da mesma forma, esse esquema ocorre em órgãos da circulação mesentérica, esplâncnica e renal, porém não de maneira contínua, pois o fluxo é regulado por mediadores associados ao volume sanguíneo e à ingestão alimentar. Nas artérias dos membros, a manutenção do fluxo diastólico é menor devido à maior resistência vascular periférica e por se tratar de artérias musculares, que têm menor conteúdo elástico em sua parede 1 . 

 O envelhecimento causa a degeneração das fibras elásticas da túnica média das grandes artérias. Microscopicamente, ocorre perda da lâmina elástica interna, fragmentação das fibras de elastina, deposição de colágeno, espessamento da média com fibrose e calcificação 8 . 

 A aterosclerose aumenta o processo de degeneração elástica da parede arterial, pois acarreta a interrupção da continuidade do endotélio, o depósito de lipídeos e a formação de mediadores inflamatórios que potencializam o desarranjo estrutural da parede vascular. Essas alterações da parede levam ao seu enrijecimento e à perda do mecanismo de controle passivo, que consiste no acolhimento do volume ejetado na sístole e no retorno elástico da parede durante a diástole. 

 O tônus da musculatura lisa da parede das artérias e a ação vasodilatadora do óxido nítrico produzido pelo endotélio são os principais elementos que influenciam ativamente a rigidez arterial. Além disso, alguns fatores como o avanço da idade e a hipertensão causam remodelamento das células musculares lisas vasculares, alterando sua expressão fenotípica de contráteis para sintéticas. Como resultado desses processos, há perda da contratilidade da parede vascular decorrente da degradação da elastina e de alterações do colágeno 7 ^,^ 9 . O óxido nítrico causa vasodilatação e relaxamento das células musculares lisas. A redução da sua disponibilidade com o envelhecimento e com a aterosclerose provoca o aumento do tônus e reduz o calibre das artérias 10 . Além desses aspectos, outros marcadores inflamatórios e produtos da oxidação contribuem para alterações celulares e extracelulares que influenciam a complacência arterial. 

## ÍNDICES DE RIGIDEZ ARTERIAL

 Após a contração ventricular, a pressão gerada na aorta navega como uma onda 7 . A velocidade da onda de pulso (VOP) consiste na razão da distância entre dois pontos do sistema arterial e o tempo gasto pela onda para percorrer essa distância ( Figura 1 ). A medida da VOP entre a artéria carótida e a artéria femoral é considerada um preditor de risco cardiovascular e é o principal indicador da rigidez arterial 1 . Em artérias com paredes rígidas, a VOP aumenta; no entanto, em aortas elásticas, isentas de doença aterosclerótica, a VOP é baixa, em torno de 3 a 5 m/s. Isso ocorre devido à distensão das paredes da aorta. Esse processo se configura como um reservatório elástico para o volume ejetado, cujo efeito denomina-se *Windkessel*, que significa, em tradução livre da língua alemã, “câmara de ar”. Esse conceito foi desenvolvido por Otto Frank, um fisiologista alemão, que também criou o modelo matemático para aplicação em grandes vasos, o qual é usado, com adaptações, até hoje 11 . Assim, o amortecimento da pressão que ocorre na aorta é responsável pela limitação do pico sistólico. Por outro lado, o recolhimento elástico é o responsável pelo aumento do fluxo durante a diástole. 

**Figura 1:** Diferença de ondas de pulso entre as artérias carótida e femoral comum e cálculo da velocidade de onda de pulso carótida-femoral. VOP: velocidade de onda de pulso; △x: distância entre dois pontos do sistema arterial; △t: tempo gasto pela onda de pulso para percorrer essa distância. Adaptado de Laurent 2 .

 Quando a pressão arterial é medida na aorta e na circulação periférica como, por exemplo, na artéria braquial, ocorrem alterações das pressões. A pressão sistólica (Psist) na aorta central é menor e aumenta à medida que a onda de pulso é transmitida para a periferia. A pressão diastólica (Pdiast) aumenta menos ou pode até diminuir 12 . Assim, a pressão de pulso (PP), que é a diferença entre a Psist e a Pdiast, aumenta nas artérias periféricas; por exemplo, entre a raiz da aorta e a artéria braquial ocorre uma amplificação da PP em torno de 14 mmHg 12 ^-^ 14 . Trata-se de um achado fisiológico chamado de amplificação da PP ( Figura 2 ). 

**Figura 2:** Amplificação da pressão de pulso. A pressão diastólica e a pressão arterial média são relativamente constantes no sistema arterial, mas, a pressão sistólica aumenta em direção à circulação periférica. Este fenômeno é chamado de amplificação da pressão de pulso e é quantificado pela razão entre a pressão sistólica periférica (PASp) e a pressão sistólica central (PASc). Adaptado de García-Espinosa et al. 14 .

 À medida que a onda de pulso navega do coração para as artérias periféricas, ocorre a amplificação da PP devido à reflexão das ondas e ao amortecimento pela viscosidade do sangue. A onda de pulso incidente encontra aumento da resistência quando chega às artérias musculares periféricas e arteríolas, causando ondas de reflexão em direção ao coração. Essas ondas de reflexão de pulso ocorrem em qualquer segmento em que há descontinuidade de fluxo, como nas bifurcações e, principalmente, quando as ondas incidentes chegam às artérias periféricas de maior resistência e menor elasticidade. Desse modo, para melhor entendimento, as ondas podem ser categorizadas como onda incidente (ou ejetora) e onda refletida; a forma da onda de pulso resultante é a somatória da onda incidente e da onda refletida ( Figura 3 ) 14 . 

**Figura 3:** Composição da onda de pulso aórtica pela onda ejetora e onda refletida. A onda de pulso aórtica é composta pelo somatório da onda ejetora e da onda refletida. PP: pressão de pulso. Pe: amplitude da onda ejetora. Pr: amplitude da onda refletida. Adaptado de García-Espinosa et al. 14 .

 A Figura 4 mostra a onda de pulso na raiz da aorta numa situação de rigidez arterial aumentada seja pelo envelhecimento ou por aterosclerose. Devido à elevação da VOP, a onda refletida chega precocemente à aorta proximal no início da sístole (ponto P1 do gráfico). A chegada precoce da onda refletida leva ao aumento da pressão arterial sistólica central, mostrado no gráfico no ponto P2. A pressão de aumentação (PAo) mede a elevação absoluta de pressão entre os dois picos sistólicos. O índice de aumentação (AIx) mede em percentual o aumento de pressão que é devido ao retorno precoce da onda refletida e é expresso pela razão entre PAo e PP multiplicada por 100. Quanto maiores as reflexões das ondas de pulso, como nos casos de elevado tônus arteriolar ou de obstruções arteriais, maior o AIx. Dessa forma, o AIx é considerado um indicador indireto de rigidez arterial e um preditor de eventos cardiovasculares 2 ^,^ 15 ^,^ 16 . Em um estudo populacional com 5.960 participantes, cada aumento do AIx de 10% foi relacionado a um risco relativo de 1,08 de aumento de chance de eventos cardiovasculares maiores, tais como infarto do miocárdio, acidente vascular cerebral (AVC) e, até mesmo, óbito 17 . 

**Figura 4:** Onda de pulso em uma aorta proximal rígida em um ciclo cardíaco. A pressão de aumentação (PAo) reflete o aumento da pressão decorrente da chegada da onda refletida durante a sístole. P1: pico sistólico atribuído à onda ejetora. P2: segundo pico sistólico atribuído ao aumento de pressão causado pela onda refletida. A incisura dicrótica corresponde à fase final da ejeção ventricular e é produzida pelo fechamento da valva aórtica. AIx: índice de aumentação. Adaptado de Husmann et al. 16 .

 Indivíduos jovens apresentam grande elasticidade das artérias. Como a VOP é baixa nesses casos, a onda de pulso refletida chega à aorta torácica na diástole. Assim, ela contribui para o aumento do fluxo diastólico e para a manutenção do fluxo constante nas coronárias e nas artérias cerebrais. Com o envelhecimento, ocorre uma elevação da VOP devido à perda da elasticidade em grandes artérias. Sendo assim, a onda refletida então passa a chegar à raiz da aorta na sístole, causando aumento do AIx ( Figura 5 ). 

**Figura 5:** Esquema gráfico das ondas ejetora e refletida na aorta proximal. (A) No jovem com artérias elásticas, a onda refletida chega na diástole, não causa aumento do pico sistólico e preserva o fluxo diastólico. **(B)** No idoso com aorta rígida, a onda refletida chega na sístole, causando aumento do pico sistólico e redução do fluxo diastólico. Adaptado de Husmann et al. 16

 A frequência cardíaca é um importante modulador da PAo. Em situações de baixa frequência, o alargamento do ciclo cardíaco e do tempo de ejeção proporcionam a chegada da onda refletida à aorta proximal durante a sístole, levando ao aumento da PAo e do AIx. Quando ocorre taquicardia, a onda de pulso refletida chega à raiz da aorta na diástole 16 . Assim, a correção do AIx pela frequência cardíaca evita a influência dessa variável na avaliação da reflexão das ondas de pulso. O AIx@75 é o AIx corrigido para a frequência cardíaca de 75 batimentos por minuto. Esse índice é fornecido pelos aparelhos de medida de rigidez arterial e é definido como AIx@75 = AIx - 0,39 × (75 – frequência cardíaca) 18 . A Tabela 1 mostra os parâmetros hemodinâmicos associados à rigidez arterial 19 . 

**Tabela 1:** Parâmetros hemodinâmicos associados à rigidez arterial (adaptado de Gajdova et al.) 19 .

**Parâmetro hemodinâmico**	**Cálculo**	**Descrição**
Pressão de pulso (PP, mmHg)	Pressão sistólica – pressão diastólica	Diferença entre as pressões sistólica e diastólica. Nas artérias periféricas, a PP pode ser até 14 mmHg maior que a pressão aórtica central.
Pressão de aumentação (PAo, mmHg)	P2 - P1	A diferença entre o segundo (P2) e o primeiro (P1) picos de pressão na onda de pulso. P1 corresponde ao pico de ejeção sistólica. P2 corresponde ao pico causado pela chegada da onda de pulso refletida.
Índice de aumentação (AIx, %)	(P2 - P1)/PP x 100	O AIx é derivado da diferença entre o segundo (P2) e o primeiro (P1) picos sistólicos, expresso como uma porcentagem da PP. Reflete a intensidade de reflexão das ondas de pulso.
Índice de aumentação corrigido para a frequência cardíaca de 75 bpm (AIx@75, %)	AIx@75 = AIx – 0.39 × (75 – frequência cardíaca)	AIx normalizado para a frequência cardíaca de 75 bpm. O aumento da frequência cardíaca reduz o AIx. Bradicardia leva ao aumento do AIx. O AIx@75 não sofre alterações com a frequência cardíaca.
Velocidade da onda de pulso (VOP, m/s)	△x/△t	Velocidade que a onda de pulso navega no sistema arterial, definida pela razão da distância entre dois pontos do sistema arterial (△x) e o tempo gasto pela onda para percorrer essa distância (△t). É o indicador de rigidez arterial mais usado devido à sua boa reprodutibilidade.

 Além da frequência cardíaca, outros fatores modulam as reflexões de onda de pulso e a pressão aórtica central. A inibição da angiotensina II, os bloqueadores de canal de cálcio e a administração de insulina reduzem as reflexões das ondas de pulso e a pressão sistólica central 20 . A resistência periférica aumentada à insulina tem efeito reverso, com aumento das ondas de reflexão 21 . 

 Nas últimas décadas, a pressão de pulso central (PPc) tem sido associada a aumento do risco cardiovascular. Tanto a PPc quanto a pressão de pulso periférica elevadas estão associadas a risco aumentado de doença coronariana em adultos de meia-idade e idosos 22 . No entanto, o risco de eventos cardiovasculares está mais associado à elevação das pressões centrais. A elevação da PPc está associada a hipertrofia do ventrículo esquerdo e a aterosclerose carotídea 15 . Em um estudo, a PPc elevada foi associada a aumento das complicações e da mortalidade em pacientes submetidos a tratamento endovascular por isquemia crítica do membro 23 . 

## REPERCUSSÕES DO AUMENTO DA RIGIDEZ ARTERIAL

 A rigidez arterial aumentada é um conceito atual e proeminente em estudos de doenças cardiovasculares, por ser um preditor de hipertensão arterial precoce e de aterosclerose. Trata-se de um biomarcador associado ao aumento da mortalidade e a danos em órgãos-alvo que resultam em doenças cardíacas, AVC e insuficiência renal. Devido a isso, pesquisas atuais têm grande interesse na investigação da rigidez arterial como um potencial alvo terapêutico para prevenir ou tratar doenças cardiovasculares. 

 Numerosos estudos têm demonstrado que o aumento da VOP está associado à elevação do risco de eventos cardiovasculares tais como doença coronariana, AVC e doença renal terminal 24 ^,^ 25 . 

 A associação da rigidez arterial com hipertensão é bem estudada. Especialmente em indivíduos jovens, a identificação de rigidez arterial aumentada é uma oportunidade para a modificação precoce de hábitos de vida e para a prevenção da deterioração irreversível dos vasos sanguíneos 25 . A análise do estudo Framingham indicou que a elevada rigidez arterial, medida pela VOP carótida-femoral, está associada à incidência de hipertensão arterial 7 anos após a medida inicial tomada pelos pesquisadores 26 . 

 A rigidez arterial aumenta a pós-carga do ventrículo esquerdo, contribui para o remodelamento ventricular e reduz sua eficiência mecânica 27 . Isso leva ao aumento da demanda miocárdica e à redução da perfusão diastólica. Assim, a rigidez arterial está associada à disfunção ventricular diastólica, a qual aumenta as pressões de enchimento e a sobrecarga dos átrios, contribuindo para hipertrofia, fibrose e fibrilação atrial. É importante destacar que a rigidez arterial está diretamente associada ao aumento do risco de insuficiência cardíaca 28 . 

 Os rins são órgãos expostos a alto fluxo sanguíneo, pois as pressões no nível do glomérulo são semelhantes às da aorta, devido às características da microvasculatura renal. A elevação da rigidez aórtica expõe o glomérulo renal a pressões excessivas, levando, com o tempo, à proteinúria e à redução da função de filtração 29 . Numerosos estudos têm mostrado a relação entre o aumento da PP e da VOP com redução da função renal e aumento da mortalidade 30 ^,^ 31 . 

 Assim como os rins, o cérebro é um órgão de alto fluxo e dependente do fluxo diastólico. A baixa resistência das artérias cerebrais facilita a penetração das ondas de pulso até a microvasculatura. Nesse caso, a transmissão de energia das ondas de pulso, aumentada em situações de rigidez arterial elevada, contribui para microinfartos cerebrais clinicamente não reconhecidos mas que, a longo prazo, podem levar a déficit cognitivo e demência 32 . Assim, a redução do fluxo diastólico que ocorre na rigidez arterial avançada contribui para o dano cerebral. Pelo mesmo mecanismo de aumento da pulsatilidade das artérias cerebrais, há associação entre rigidez aórtica e acidente vascular hemorrágico 33 . 

## MÉTODOS DE MEDIDA DOS ÍNDICES DE RIGIDEZ ARTERIAL

 É possível medir a VOP por meio de cateteres na aorta; porém, a medida invasiva tem pouca aplicabilidade prática. Estudos com medidas de pressão central são realizados em humanos somente em situações de pesquisa clínica e para validação dos métodos não invasivos 1 . 

 A medida da rigidez arterial e a inferência dos valores de pressão central são efetuadas por meio de modelos matemáticos. Os equipamentos utilizados para determinar as medidas dos índices de rigidez são classificados em quatro categorias: equipamentos que usam um tonômetro para medir a onda de pulso, equipamentos com manguitos que captam a onda de pulso por oscilometria, medidas com uso do ultrassom e medidas com uso de ressonância nuclear magnética (RNM). 

### Equipamentos que usam tonômetro para medida da onda de pulso

 O SphygmoCor® (AtCor Medical, West Ryde, Austrália) é um equipamento bastante usado em pesquisas para medida da VOP. Esse instrumento utiliza uma sonda que mede a pressão (tonômetro) em dois locais, normalmente sobre a carótida cervical e sobre a artéria femoral no nível inguinal, onde o pulso é detectável. O aparelho é sincronizado com o eletrocardiograma, para a medição das ondas de pulso durante o ciclo cardíaco. A distância é medida manualmente do ângulo supraesternal até a artéria femoral 2 . Outros equipamentos validados para a medição da onda de pulso baseada em tonometria são o Complior® (ALAM Medical, Vincennes, França) e o PulsePen® (DiaTecne, Milão, Itália) 1 . 

###  Equipamentos com manguitos que captam a onda de pulso por oscilometria 

 O Mobil-O-Graph ® (IEM, Stolberg, Alemanha) estima a pressão aórtica central e a VOP por meio de um algoritmo obtido a partir de medidas na artéria braquial. Após a medida da pressão arterial, o manguito é insuflado por aproximadamente 10 segundos no nível da pressão diastólica para a captação das ondas de pulso. Assim, por meio de um modelo matemático, uma série de parâmetros hemodinâmicos são estimados, incluindo as medidas das ondas de reflexão, da pressão e do AIx@75. O aparelho foi validado para a medida da VOP em comparação com exames invasivos e não invasivos 34 ^,^ 35 . É importante apontar que equipamentos oscilométricos portáteis têm a vantagem de serem menos dependentes de operadores e mais fáceis de utilizar. Outros equipamentos oscilométricos são o VP1000® (Omron Healthcare, Kyoto, Japão) e o Vasera® (Fukuda Denshi, Tóquio, Japão) 1 . 

### Medidas com ultrassom

 A distensibilidade das artérias e as características das curvas de fluxo podem ser acessadas facilmente na carótida cervical, na artéria braquial e na artéria femoral, na região inguinal. Alguns programas computacionais destinados à medida da distensão dos vasos foram criados para possibilitar o cálculo da PP e a estimativa das pressões centrais (exemplo, ARTLAB®, Esaote, Gênova, Itália). A VOP pode ser medida por meio de dois transdutores, um sobre a artéria carótida e outro no abdômen, sobre a aorta, ou sobre a artéria femoral. Nesse caso, há a necessidade de o aparelho de ultrassom estar conectado ao eletrocardiograma para a adequada sincronização com o ciclo cardíaco. Por esses motivos, esse método é considerado pouco prático, sendo raramente utilizado em pesquisa clínica 1 . 

### Medidas com uso de ressonância nuclear magnética

 Assim como no ultrassom, a RNM pode ser usada para medir a distensibilidade das artérias de acordo com o ciclo cardíaco, com a vantagem de poder acessar os vasos torácicos. A RNM com meio de contraste endovenoso pode ser usada para medir a velocidade do fluxo e a VOP. Entretanto, o custo do método e a dificuldade relacionada a operadores treinados para manejá-la dificultam sua aplicabilidade clínica 1 . 

##  RELAÇÃO DA DOENÇA ARTERIAL PERIFÉRICA COM RIGIDEZ ARTERIAL 

 A DAP na sua forma avançada, a isquemia crítica, pode ser considerada um estágio terminal de rigidez arterial aumentada 16 . É bem conhecido que o ITB é um marcador independente de mortalidade e morbidade; porém, sua relação com índices de rigidez arterial é pouco estudada 36 . 

 A rigidez aórtica aumentada está associada ao desenvolvimento de aterosclerose como resultado de alterações do fluxo em grandes artérias e de forças de cisalhamento durante a sístole 1 ^,^ 26 . A rigidez da parede da aorta provoca a redução do gradiente de impedância (resistência ao fluxo sanguíneo) entre a aorta complacente e a circulação periférica composta por artérias musculares. Devido à redução da complacência da aorta, ocorre transmissão em excesso das ondas de pressão para a microvasculatura, causando as lesões em órgãos-alvo 26 ^,^ 32 . 

 A rigidez arterial aumentada está associada à redução da distância de marcha em claudicantes 37 . Embora haja várias publicações voltadas para os índices de rigidez arterial em pacientes claudicantes 38 ^-^ 42 , há poucas investigações em pacientes com isquemia crítica do membro e, mesmo assim, os achados são inconclusivos. Em casos de obstruções aorto-ilíacas graves, a VOP medida na artéria femoral mostra-se reduzida 43 . Em contrapartida, a magnitude da reflexão das ondas de pulso, medida pelo AIx, está aumentada nos pacientes com doença arterial periférica 44 ^,^ 45 . 

 As obstruções arteriais ao longo dos segmentos aorto-ilíaco e infrainguinal são pontos de reflexão precoce das ondas de pulso; sendo assim, o retorno precoce à raiz da aorta durante a sístole aumenta a pós-carga do ventrículo esquerdo e reduz a perfusão miocárdica 16 ^,^ 25 . A relação entre demanda e suprimento miocárdico desfavoravelmente alterada devido ao aumento da rigidez arterial é um dos fatores que aumenta a mortalidade nos pacientes com DAP 36 . 

 As principais causas de mortalidade nos pacientes com DAP avançada são doença cardíaca isquêmica, AVC e mobilidade reduzida devido a amputações maiores e menores. Há preditores estabelecidos para esses desfechos: idade avançada, diabetes e redução do ITB 16 . É possível que indicadores de rigidez arterial como a VOP e o AIx possam ser preditores de desfechos clínicos na DAP; porém, essa relação ainda não foi estudada. Conhecer os parâmetros hemodinâmicos na DAP avançada pode ajudar a entender a relação desses parâmetros com a mortalidade por doença coronariana e cerebral nos pacientes com DAP. 

## CONCLUSÃO

 Considerando os pacientes com DAP, é importante identificar aqueles com risco mais elevado. É possível que a rigidez arterial aumentada possa ser uma fator que leve ao aumento da mortalidade em pacientes claudicantes ou com isquemia crítica. Da mesma forma, os índices de rigidez arterial podem estar associados a desfechos como preservação do membro ou amputações maiores. Essas associações ainda não foram estudadas. Uma vez estabelecida a relação entre o grau de isquemia do membro e os índices de rigidez arterial, estes poderão ser utilizados em estudos longitudinais como fatores prognósticos dos desfechos da DAP.
